# Potential Role of Diabetes Mellitus-Associated T Cell Senescence in Epithelial Ovarian Cancer Omental Metastasis

**DOI:** 10.3390/life11080788

**Published:** 2021-08-04

**Authors:** Rhianne Broadway, Nikita M. Patel, Lucy E. Hillier, Amal El-Briri, Yulia S. Korneva, Dmitry A. Zinovkin, Md Zahidul I. Pranjol

**Affiliations:** 1 School of Life Sciences, University of Sussex, Falmer, Brighton BN1 9QG, UK; rb577@sussex.ac.uk (R.B.); leh46@sussex.ac.uk (L.E.H.); 2 William Harvey Research Institute, Barts and the London School of Medicine and Dentistry, Queen Mary, University of London, London EC1M 6BQ, UK; n.m.patel@qmul.ac.uk (N.M.P.); aelbriri@hotmail.co.uk (A.E.-B.); 3 Department of Pathological Anatomy, Smolensk State Medical University, Krupskoy St., 28, 214019 Smolensk, Russia; ksu1546@yandex.ru; 4 Smolensk Regional Institute of Pathology, Gagarina av, 214020 Smolensk, Russia; 5 Department of Pathology, Gomel State Medical University, 246000 Gomel Region, Belarus; zinovkin2012@gmail.com

**Keywords:** epithelial ovarian cancer, milky spots, senescence, T cells, diabetes mellitus

## Abstract

Epithelial ovarian cancer (EOC) is one of the most common causes of cancer-related deaths among women and is associated with age and age-related diseases. With increasing evidence of risks associated with metabolic inflammatory conditions, such as obesity and type 2 diabetes mellitus (T2DM), it is important to understand the complex pathophysiological mechanisms underlying cancer progression and metastasis. Age-related conditions can lead to both genotypic and phenotypic immune function alterations, such as induction of senescence, which can contribute to disease progression. Immune senescence is a common phenomenon in the ageing population, which is now known to play a role in multiple diseases, often detrimentally. EOC progression and metastasis, with the highest rates in the 75–79 age group in women, have been shown to be influenced by immune cells within the “milky spots” or immune clusters of the omentum. As T2DM has been reported to cause T cell senescence in both prediabetic and diabetic patients, there is a possibility that poor prognosis in EOC patients with T2DM is partly due to the accumulation of senescent T cells in the omentum. In this review, we explore this hypothesis with recent findings, potential therapeutic approaches, and future directions.

## 1. Introduction

Epithelial ovarian cancer (EOC) is the eighth most common cause of cancer-related deaths among women globally, with a survival rate of less than 50% and an average age at diagnosis of 63 years [1,2]. The poor prognosis of ovarian cancers, which lack symptoms at an early stage, means that 80% of all cases are diagnosed at a later, more advanced stage [1,3]. With age and age-related disorders, such as diabetes, acting as significant risk factors for disease progression in EOC, it raises the question of which element of the ageing process is leading to such a change in prognosis [2]. With many of the components of the omentum changing with age, including a loss of the basement membrane, which aids tumour invasion, and distinct changes to mesothelial cells, the peritoneal extracellular matrix, immune cells, and adipocytes [2], a concept that seems to be key to increased inflammation associated with ageing is termed cellular senescence. Accumulation of senescent cells in age-related disease, such as diabetes, is known to create a proinflammatory environment for disease progression [2,4]. Nevertheless, the recent discovery of increased numbers of senescent T cells in type 2 diabetic patients may indicate a role of senescent immune cells as potential contributors to the progression and advancements in EOC metastasis [5].

A number of risk factors and disorders, such as ageing, smoking, late pregnancy, diabetes, and obesity, have been reported to be associated with poor survival of ovarian cancer patients. For instance, in recent years, type 2 diabetes mellitus (T2DM) has been identified as an independent risk factor for mortality in patients with EOC. Bakhru et al. (2011), in their observational study of 642 patients, revealed a decrease in overall survival (OS) from 6 years for nondiabetic patients to 4 years for diabetic patients with EOC [6]. A retrospective cohort study (n = 367) confirmed these findings, with diabetic patients exhibiting both poorer progression-free survival (PFS) (10.3 vs. 16.3 months) and OS (26.1 vs. 42.2 months) [7]. The association between T2DM and EOC is complex as there are multifactorial aspects, but also the molecular basis of this remains unclear. Due to these pathophysiological features, diabetes is associated with a chronic, low-grade systemic inflammation, which is believed to be linked with cellular senescence, particularly in the lymphocytes and macrophages [8,9,10]. According to early studies, this persistent low-grade inflammation in T2DM was believed to be caused by macrophages, which led many groups to concentrate on the role of innate immunity in disease development. However, now it has been discovered that the number of senescent T cells (both CD4^+^ and CD8^+^) increases in patients with T2DM and is believed to be a contributory factor to disease progression [11].

Another condition that contributes to EOC progression is obesity. One of the biggest studies, “The Million Women Study” based in the United Kingdom, followed 1.2 million women for an average of 5.4 years for cancer incidence and 7.0 years for cancer mortality. This study found that women with a BMI ≥ 25 have a higher incidence of EOC compared with their normal weight counterparts/women with a BMI of 18.5–24.9 [12]. Nagle et al. (2015) studied the associations between histopathological types of EOC and disease outcomes in patients with obesity, showing increased mortality in cases of high- and low-grade serous and endometrioid cancers [13]. In ovarian cancer patients, obesity induces chronic inflammation that can modify the tumour microenvironment and induce T cell senescence [14,15,16], which could potentially contribute to EOC progression. Chronic inflammation has been shown to involve a chemokine network that influences the migration and invasion of cancer cells, which support the tumour microenvironment for cancer progression by increasing the inflammatory burden [17,18]. This chronic inflammation has been strongly associated with inducing senescence, particularly immune senescence in age-related metabolic disorders, such as T2DM, and in ovarian cancer [11,19]. Given that chronic-inflammation-induced senescence is a detrimental factor associated with poor prognosis in T2DM and obesity comorbidities, there is a possibility that these immune cells in the ageing peritoneum of T2DM patients contribute to creating a more proinflammatory, tumour-friendly premetastatic niche for migrating EOC cells to form secondary foci. Subsequently, this cascade could lead to metastasis. In this review, we will explore this hypothesis in the light of immune senescence in “milky spots” and secondary ovarian tumour growth and metastasis in the omentum.

## 2. Omental Milky Spots as the Preferential Site for Colonisation by Epithelial Cancer Cells

**Figure 1:**
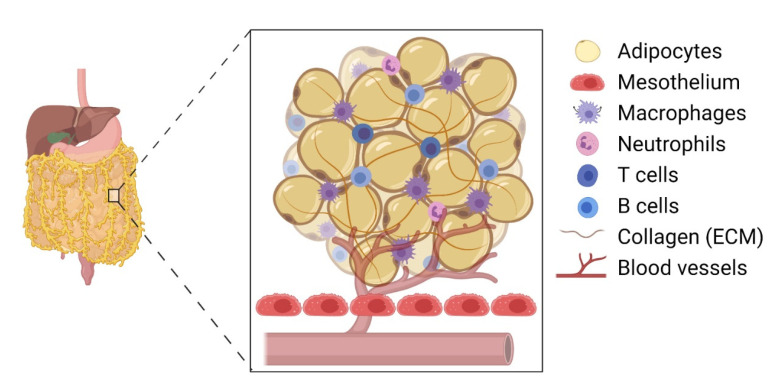
Immune-related cellular structure of the omentum. The omentum predominantly consists of adipocytes within a mesh of extracellular matrix proteins, such as collagen. Immune cells such as macrophages, T cells, B cells, and neutrophils are found in clusters and dispersed within the omentum. The adipocytes are lined with mesothelial cells, which are the first point of contact for migrating cancer cells. The omentum is highly vascularised, which significantly aids in cancer progression.

The omentum is the preferred site for peritoneal cancer cells to colonise and proliferate [3] and is the most common site of EOC metastasis [1] (Figure 1). The omentum is highly vascularised and formed as the parietal peritoneum folds, lying between the parietal peritoneum and the anterior surface of the abdominal organs. Structurally, the omentum has a matrix that is rich in collagen with a mesothelial cell layer and a thin basement membrane [2]. It shows two contrasting structural regions—one with a collagenous membranelike organisation and the other rich in adipose tissue—accommodating blood vessels, lymph nodes, and clusters of immune cells [20]. The healthy omentum plays a key role in repairing injury, fighting infections, and releasing factors to aid local homeostasis [3]. It is also known to have a significant impact on peritoneal immunity due to the high number of lymphoid clusters or “milky spots” present [2].

EOC cells disseminate from the ovary into the peritoneal fluid. With physiological movement, the tumour cells primarily attach to the omentum via alpha and beta integrin interaction with the omental surface mesothelial cells [3]. This initial interaction allows for the entry into the underlying matrix via the proteolytic degradation of extracellular matrix (ECM) proteins by cancer-cell-secreted matrix metalloproteinases (MMPs) [21,22]. For instance, MMP2 lyses collagen IV of the omentum, causing a loss of the basement membrane, which increases with age [2]. Through a cycle of tumour cell proliferation, migration, and invasion, metastatic lesions are formed within the omentum. An angiogenic switch takes place in response to growth factors secreted by a tumour and other local cells, which initiates metastasis. Subsequently, the tumour begins the formation of new vessels in order to increase blood supply [23,24,25,26,27,28]. Prior to these new vessels forming, the tumour is dependent on the existing blood supply within the omental microenvironment.

Despite being predominantly composed of adipose tissue, the omentum is an active immune organ with clusters of immune cells found across the tissue [29]. These milky spots, or fat-associated lymphoid clusters (FALCs), play an important role in inducing an immune response to tissue insult, such as injury or infection. These were first seen as dense corpuscles in the omentum by Recklinghausen in 1863 [30] and, in 1993, were reported to be the most prominent site of malignant cell implantation in the peritoneal dissemination of mice [31,32]. Forming during 20–35 weeks of gestation, they consist principally of B (B1 and B2) and T lymphocytes with macrophages, plasma cells, lymphatic vessels, and a blood supply also present [3,20]. The milky spots have compositions that can be split into structural, migratory, and functional elements, where the former is composed of fibroblasts, adipocytes, mesothelia, and endothelia with lymphocytes, granulocytes, and monocytes making up the migratory region. Macrophages, stromal cells, and the endothelium of the veins form the functional element [33]. Due to their lack of any form of capsule, the contents of the milky spot are in direct contact with the surrounding adipose tissue, allowing them to utilise the abundance of blood vessels and collect antigens or pathogens from the peritoneal fluid directly [34]. This supports their function in the healthy omentum as they are able to recognise antigens and pathogens from the peritoneal cavity and aid in processing them through inflammatory regulation [4].

Clark et al. (2013) provided compelling evidence that EOC cells preferentially colonise the milky spots in the omental tissue compared with other peritoneal tissues [35]. In their experiment, both C57BL/6 and nude mice were injected (intraperitoneally) with EOC cell lines, such as ID8, SKOV3, CaOV3, and HeyA8 cells. Interestingly, their histological analysis revealed 48 foci of cancer cells within the omentum and only 5 foci in the splenoportal fat [35]. There were no ovarian cancer cells detected in the uterine or gonadal fat, indicating a milky-spot-specific directed migration of EOC cells within the peritoneum. For example, cancer cell migration increased by 95-fold in the presence of omentum-tissue-conditioned media. More specifically, the presence of adipose tissue containing no milky spots revealed a 75% reduction in cancer cell migration, suggesting a key role of immune cells within the milky spots inducing a directed migration of these cells [35]. However, other cells, such as mesothelial cells and cancer-associated adipocytes, may also play a role [36].

In the early stage of tumour growth within the premalignant omentum, immune cells in the milky spots secrete protumourigenic factors, establishing a further crosstalk between tumour cells and local cancer-associated cells. This is further aided by hypoxia, resulting in the cancer cell migration, proliferation, and formation of secondary foci [35]. These micrometastatic proliferative tumour cells then aggregate and disrupt the structure of the milky spots, leaving immune cells dispersed within the tumour mass, suggesting that these resident immune cells in the milky spots aid tumour invasion and metastasis [37]. A study by Etzerodt et al. (2020) involving CD163^−^ and Tim4^−^ expressing macrophages showed the promotion of malignant ovarian cancer progression in the omentum via macrophage and tumour cell association [1]. For instance, the depletion of CD163^+^Tim4^+^ macrophages prevented metastatic disease development and reduced ascite formation [1]. The secretion of CCL6 and CCL23 by omental macrophages significantly increased the migration and colonisation of EOC cells via binding to CCR1 receptors on cancer cells and inducing the activation of the ERK1/2 and AKT pathways, where ERK activation is pivotal in cancer cell survival through the upregulation of antiapoptotic proteins and the inhibition of caspase activity [38,39]. For instance, a reduction in omental macrophages caused a diminished level of EOC cell colonisation in the omentum, as did the inhibition of CCL6, highlighting that these macrophages possess a key role in tumour progression [39].

It has been found that the number of milky spots in the omentum increases in the presence of inflammatory cues/stimuli. For example, intraperitoneal injection of lipid A, a component of bacterial polysaccharide, increased the number of macrophages in the omentum [40]. Additionally, the number and size of milky spots increased in response to intraperitoneal injection of polydextran particles or polyacrylamide beads [41,42]. Interestingly, the omentum produced a CXCL13-mediated response, which is produced by CD4 T cells, and supported T cell-dependent B cell responses, including isotype switching, somatic hypermutation, and limited affinity maturation [43,44]. Tumour-associated macrophages have also been suggested to influence T cells by altering their cytotoxic function and recruitment in a cancer microenvironment in aged mice (reviewed in [45]). Moreover, the omentum also recruited effector T cells and produced CD4^+^ and CD8^+^ T cell responses to peritoneal antigens, suggesting that the adaptive immune system is a key component in inflammatory conditions, such as a growing tumour [44].

## 3. Diabetes Mellitus-Associated T Cell Senescence

The induction of T cell senescence was reported several years ago, although its disease-associated roles are now becoming more evident. T cell senescence is characterised by a reduction in the total naïve T cell pool, being replaced with differentiated T cells, such as effector memory (EM) T cells and terminally differentiated effector memory (EMRA) T cells [5,46]. The age and status of T cells are tracked by their cell-surface markers, as they do not express the costimulatory molecules CD27 and CD28, but do express CD57 and KLRG1, both of which function to reduce the proliferative capacity [46]. Senescent CD8^+^ T cells have recently been shown to increase in abundance in older donors with a twofold increase compared with younger donors in their early 20s, suggesting their major contribution to a reduced immune function and enhanced susceptibility to disease [47].

Metabolic disorders, such as obesity and T2DM, have also been shown to induce cellular senescence through an accumulation of these cells in organs as well as in the circulation [48]. For instance, Lau et al. reported an increase in EM and EMRA CD45RA re-expressing T cells in T2DM patients [11]. Overall, the authors showed that T2DM acted as a driving factor in the premature ageing of both CD4^+^ and CD8^+^ cells, increasing T cell differentiation and senescence, which contributed to systemic inflammation, as detected by raised levels of neutrophils [11]. Moreover, these senescent T cells expressed significantly higher numbers of the CX3CR1 chemokine receptor, indicating a promigratory phenotype that is seen during the early stages of tumour progression [11,49,50].

## 4. Age-Related Immunosenescence and Cancer

The presence or absence of T cells infiltrating (TIL) the tumour in EOC patients has a significant impact on survival. These cells inhibit cancer cell growth by secreting antiangiogenic cytokines, which have been shown to be increased in ovarian tumours with TILs present [51,52]. However, with ageing, CD4^+^ cells secrete less IL-2 in comparison with their younger counterparts [53], and their memory capabilities are also decreased [54]. A reduction in CD8^+^ antitumour capabilities is also seen, promoting tumour growth and development [55]. Therefore, all these factors lead to the aged TIL subset being inactive when compared with its younger counterpart and having a reduced antitumourigenic effect. Moreover, senescent cells accumulate and can also be found within persistent and chronic infections, where the immune system is repeatedly trying to control the infection [46]. This age-related change in immune physiology in T2DM patients who are diagnosed with EOC can potentially contribute to cancer progression. This change is more likely to take place among T cells due to their high proliferative status, which are thus inclined to become senescent. However, T cells can remain viable with subsets that are resistant to programmed cell death [56].

In humans, the T lymphocyte progeny expresses the cell marker CD28, but in the ageing population, there is an increase in circulating CD8^+^ CD28^null^ T cells [56]. CD8^+^ T cells are relatively more susceptible to senescence as these cells have a single β-bound protein, the CD28 promoter complex [56]. Loss of CD27 is another marker of immunosenescence, indicating the intolerance to proliferate in some subsets of senescent T cells [56]. Additionally, the expressions of CD45RA and CD57 (rather than the loss of CD28) are markers of senescent T cells [57,58]. Although there are no morphological changes in the subsets of senescent T cells, CD8^+^CD28^−^CD57^+^ T cells display a higher expression of proinflammatory cytokines than other subsets under resting conditions [59]. Another distinguishable feature is the inherent mitochondrial metabolic capacity that governs the stability of CD4^+^ and CD8^+^ T cell senescence [60]. Nevertheless, senescent immune cells have been linked to an arsenal of physiological and pathological manifestations, including pulmonary diseases, autoimmune diseases, allogeneic transplantation, and various cancers [57,61,62,63,64,65].

Many studies have shown that T cells that have developed a senescent phenotype are considered to have reached a critical phase known as clonal exhaustion, defined by proliferative incompetence. However, further research has shown that this is not true for all senescent T cells [57]. Senescent cells in general express permanent cell cycle arrest, and it is widely accepted that repeated antigenic activation drives senescence in T cells [57]. Nonetheless, recent findings have shown that senescent T cells can evade cell cycle arrest by upregulating telomerase activity, allowing these CD8^+^ CD28^null^ cells to proliferate under optimum conditions [57]. The senescent T cells expressing CD8^+^CD28^−^ (CD27^+^) are not completely senescent due to their ability to activate telomerase gene expression and permit cellular proliferation. This illustrates that the presence of CD27 is an indication for T cells, which are not completely senescent but are close to terminal differentiation [57]. Experimental evidence shows that CD8^+^ CD28^−^ cells expressing CD27^−^ have shorter telomeres with reduced proliferative capacity, as the costimulation can no longer induce cellular proliferation [66].

Not only do CD8^+^ CD28^−^ T cells have an enhanced ability to proliferate despite the expression of CD28^−^ that signifies senescence, but also a population of senescent T cells (CD27^−^) exhibits apoptotic resistance, promoting their differentiation to become late-differentiated CD8^+^ CD28^−^ T cells [57]. However, not all senescent T cells express this apoptotic resistance potential, whereby CD8^+^ CD28^−^ CD57^+^ T cells are cleared following antigen stimulation [57]. Chronic inflammation results in the replacement of these cells by newly formed CD8^+^ CD28^−^ (CD57^+^) cells. Increased circulating senescent immune cells, particularly CD8^+^CD57^+^ (or CD8^+^CD28^null^) T cells, have been linked to critical roles in other diseases, including hypertension and chronic kidney disease, as well as T2DM [67,68]. The increase in circulating senescent immune cells is a phenotype solely observed in humans and primates, as in aged mice, this is not detected, and the reasons for this remain unknown [56]. Moreover, a cohort study suggested that there is a case of premature senescence in children with chronic kidney disease as the T cell senescence markers CD28^null^ and CD57^+^ were significantly increased relative to controls [69]. Whether these senescent T cells have increased cytokine secretion was not investigated [69]. Nevertheless, apoptotic resistance governs the differentiation and proliferation of intermediate senescent T cells to generate T cells that are at a stage of complete cellular senescence [57]. This phenotype is pathological in that it allows the accumulation of senescent T cells over a long period of time [57], which may be implicated in T2DM-associated chronic inflammation.

Although they lose their proliferative capacity, senescent T cells remain metabolically active, releasing a pool of both pro- and anti-inflammatory cytokines, known as the senescence-associated secretory phenotype (SASP) [5]. CD8^+^ T cells that have undergone SASP have a critical change in function, with reduced cytotoxic capacity and downregulation of its effector molecules, perforin and granzyme B. Instead, the cells have a very strong inflammatory profile, recruiting other immune cells to the site for clearance through immune surveillance [70,71]. The SASP profile of T cells and their reduced cytotoxic abilities can contribute to the establishment, growth, and maintenance of cancer, and increased levels of CD8^+^ senescent T cells can be found in certain cancer types [70]. Regulatory T cells (Tregs) and tumour Tregs can themselves induce senescence in effector and naïve T cells as they compete for resources, causing immunosuppression within the tumour microenvironment and a reduced immune response [70]. SASP components, such as growth-regulated oncogene-α, have been shown to cause epithelial cell proliferation in various types of cancer, including breast and prostate [72].

**Figure 2:**
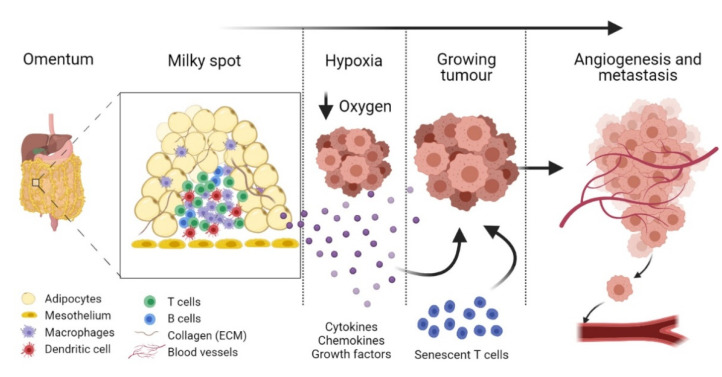
Immune cells within the milky spots could aid in tumour progression and metastasis. In the hypoxic tumour microenvironment, immune cells secrete inflammatory cytokines, chemokines, and growth factors, which enhance tumour proliferation and migration, leading to omental invasion. In type 2 diabetes mellitus (T2DM), a low-grade chronic inflammation results in T cell senescence, which contributes to tumour progression by inducing a senescence-associated secretory phenotype (SASP) that consists of protumourigenic factors. These factors, along with tumour cell secretion, result in a complex crosstalk between local microvasculature and growing cancer cells, leading to angiogenesis and, consequently, metastasis.

The most common form of ovarian cancer arises from epithelial cells [73]. Therefore, after the invasion of the ovarian cancer to within the milky spots, it is possible that the senescent T cells contribute to the proliferation and growth of the EOC cells rather than the removal by immune surveillance. Other factors secreted by senescent cells promote angiogenesis, such as vascular endothelial growth factor (VEGF) and angiogenin [72]. Therefore, the already highly vascularised environment and the SASP of senescent T cells possibly work in synergy to allow tumour colonisation and growth to ensue, as the blood supply, and thus the nutrient supply, within the milky spot is increased by both of these factors (Figure 2). IL-1, another SASP component, not only is instrumental to angiogenesis but also causes increased vascular permeability, and hence can contribute to the metastasis of the cancer from the primary site [72,74]. Within the milky spots, this could then account for the rapid metastasis into other sites in the body and surrounding tissue. Other secreted SASP factors, such as colony-stimulating factor (CSF) and VEGF, recruit further immune cells into the tissue, including monocytes that undergo differentiation into tumour-derived macrophages. These macrophages then promote and reinforce the inflammatory response and increase angiogenesis [3,73]. Table 1 summarises the functions of selected SASPs in tumour progression, angiogenesis, and immunosuppression.

## 5. Could Premature Immunosenescence Contribute to EOC Progression in DM?

It was recently reported that T cell senescence appears in prediabetics, with increased expression of proinflammatory cytokines (TNF-α and IL-6) and cytotoxic enzymes (granzyme B) in CD28^−^CD57^+^CD8^+^ T cells [59]. Additionally, the levels of TNF-α and perforin were significantly increased in prediabetic patients, despite no increases in the CD4^+^ T cell number being observed in prediabetics compared with control subjects [59]. This indicates that the accumulation of senescent T cells can occur earlier in life, resulting in a more chronic proinflammatory environment. Moreover, premature ageing of T cells has been reported in T2DM and prediabetic patients [11,59]. This may indicate that in EOC patients, where age and T2DM are detrimental factors, early appearance and accumulation of immune senescence may contribute to a low-grade chronic inflammation in the peritoneum [102], specifically within and surrounding the milky spots. This could influence the formation of a premetastatic niche and lead to a poor prognosis of the disease. Therefore, further investigation is required to identify novel biomarkers for stratifying early-stage ovarian cancer patients suffering from T2DM [103] and to strategise and manage preventative measures, as early diagnosis in EOC patients is rare.

Nevertheless, research needs to be conducted to fully establish the link between senescent T cells in age, age-related diseases, chronic infections, and their role within the growth and metastasis of EOCs. Since early diagnosis of EOC is rare with a high incidence rate, and its risk is associated with senescence-inducing metabolic disorders, such as T2DM, efforts should be in place to detect immunosenescence earlier in life. Early diagnosis could lead to preventative therapeutic interventions. Currently, senolytic medications are known to selectively clear senescent cells and have been shown to both increase life span and alleviate age-related cardiovascular disease in mice [104,105]. Senolytics have also been demonstrated to decrease the population of senescent cells in human trials, while decreasing the numbers of SASP factors found within the blood [105,106]. Moreover, chimeric antigen receptor (CAR) T cells have been developed as a senolytic agent, which could identify markers and selectively target and destroy senescent cells [107]. This could be developed further to target to kill senescent T cells or to reverse exhaustion to switch CD8^+^ T cells to an antitumourigenic state [108]. Moreover, metformin, an antidiabetic drug, has shown some promises in alleviating cellular senescence and demonstrated preclinical anticancer, antiangiogenic effects in ovarian cancer; however, it lacks clinical evidence as an anticancer agent in ovarian cancer patients [109,110]. Thus, there is a possibility to clear senescent immune cells through an individual or a combination of treatment strategies within the milky spots and the omentum in disease states, such as T2DM and chronic infection. Further research is required to overcome barriers posed by solid tumours and their responses to immune surveillance and treatments, including immunotherapies [111].

## 6. Conclusions

Type 2 diabetes mellitus (T2DM), a risk factor for epithelial ovarian cancer (EOC), is a chronic metabolic and inflammatory disorder that results in immune senescence. Although immune cells, such as T lymphocytes, remain the body’s main cancer surveillance system, induction of senescence in these cells can reverse the antitumourigenic immunity contributing to the systemic low-grade inflammation found in T2DM. Evidence shows that senescence of T cells occurs in prediabetic patients, which could accumulate within the omentum in a detrimental capacity, with the omentum already favouring a metastatic niche for EOC. Their secretion of a senescence-associated secretory phenotype (SASP) and crosstalk with omental cells could potentially result in a low-grade chronic inflammatory environment, aiding in creating a premetastatic niche, therefore encouraging the metastasis in EOC. Thus, detecting immune senescence and preventing its damaging effect on the omentum, within both the aging population and age-related diseases, could potentially reduce the risk of ovarian cancer progression and improve prognosis. Advances in senolytics and immunotherapies could give rise to the possibility of EOC cancer prevention if proinflammatory diseases or high levels of immune senescence in the aging population are identified at an earlier stage, reducing disease burden. Therefore, it is of importance to determine the true role of immune senescence in patients with EOC and T2DM, allowing improved health, diagnosis, and earlier intervention. Understanding the roles senescence plays in age-related diseases and EOC will allow for further developments in diagnosis and treatment.

## Figures and Tables

**Table 1:** Genes identified from the T cell SASP [5] and their role in tumourigenesis.

T Cell SASP	Cell Proliferation	Cell Migration	Tumour Invasion	Angiogenesis	Immune Suppression	Ref.
CCL5	↑	↑	↑	↑	↑	[75,76,77,78,79,80,81,82,83,84,85,86,87,88,89,90]
CCL16	n.d.	↑	n.d.	↑	n.d.	[91,92]
CCL23	n.d.	↑	n.d.	↑	↑	[93,94,95]
IL-18	↓	n.d.	n.d.	↓	n.d.	[96,97]
PDGF-D	↑	n.d.	↑	↑	n.d.	[98,99,100,101]
TNF-α	↑	n.d.	↑	↑	n.d.	[78,79,81]

CCL5, 16, 23: C-C motif chemokine ligand 5, 16, 23; IL-18: interleukin-18; PDGF-D: platelet-derived growth factor-D; TNF-α: tumour necrosis factor-alpha; ↑: increase; ↓: decrease; n.d.: not determined.

## Data Availability

Not Applicable.
